# Optimization and characterization of pectin extracted from banana and papaya mixed peels using response surface methodology

**DOI:** 10.1002/fsn3.2754

**Published:** 2022-01-23

**Authors:** Tanje Mada, Ramesh Duraisamy, Fisseha Guesh

**Affiliations:** ^1^ 128157 Department of Chemistry (Food and Sugar Technology) Arba Minch University Arba Minch Ethiopia; ^2^ 128157 Department of Chemistry College of Natural Sciences Arba Minch University Arba Minch Ethiopia

**Keywords:** anhydrouronic acid, banana, microwave extraction, papaya, pectin

## Abstract

A massive amount of fruit peels generated from fruit processing industries and household kitchens has resulted in nutritional loss and environmental problems. Pectin is a polysaccharide that is isolated from fruit peels and has been attributed to various applications. By proper waste management practices and the use of efficient methods for retrieval of pectin from fruit, peels would benefit from resource management. This study has aimed at the extraction of pectin from locally available fruit peels. Pectin extraction from banana–papaya peel was done by microwave‐assisted extraction. The influence of temperature, time, and pH on extraction yield and anhydrouronic acid content was analyzed using software Design Expert 11. The optimum operating conditions such as temperature, time, and pH to achieve maximum yield (23.74%) and anhydrouronic acid (69.97%) were determined as 73°C, pH 2, and 35 min, respectively. Physicochemical assets of the extracted pectin, such as moisture, ash, protein, methoxyl content, degree of esterification, equivalent weight, and acetyl value, were determined as 7.2 ± 0.27%, 6.20 ± 1.26%, 3.92 ± 0.05% 8.37 ± 0.42%, 67.91 ± 0.33%, 783.69 ± 0.46 g/mol, and 0.48 ± 0.11%, respectively, and some functional properties like water absorption capacity, oil absorption capacity, swelling capacity, and emulsifying activity and emulsion stability are found as 8.23%, 18.44%, 22.73%, 45.16%, and 29.33%, respectively.

## Graphical abstract

The current study focused on the extraction and optimization of pectin from banana–papaya mixed peel wastes. The extraction conditions were optimized by the analysis of variance (ANOVA) and response surface methodology (RSM) approach. The physicochemical and functional properties of the obtained pectin were also evaluated.

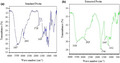

## 1 INTRODUCTION

Recently, fruits and vegetables are increased significantly due to the growing population and changing of eating propensities; more individuals shifting to vegetarian‐based diets (Malenica & Bhat, 2020; Kumar et al., 2020). The processing condition of fruits and vegetables creates wastes that constitute around 25%–30% of whole fruits (Sagar et al., 2018). These wastes need to be converted into useful products; hence, there is a need to look at the asset of recycling and production of useful ingredients (Khamsucharit et al., 2018). The wastes are composed majorly of seed, skin, peel, and pomace containing more sources of bioactive compounds, fibers, and others, which are advantageous for human well‐being (Malenica & Bhat, 2020).

Pectin is a heteropolysaccharide that is present in the primary cell walls of the fruit and vegetables, and the center lamella of the plant‐cell wall contains 1,4‐linked α‐galacturonic acid residues (Begum, 2014). It is mainly existent in fruits and vegetables and is found in around 35%–40% of the primary cell wall in all dicot plants (Vanitha & Khan, 2019). It is a food value‐added substance or food additive stated by the joint FAO/WHO Expert Committee; European Commission recommends that pectin requires to comprise of at least 65% of galacturonic acid which meets the criteria for food additive pectin (Müller, 2016). The degree of esterification (DE) rate may be found as >50%, which is grouped as high methyl ester pectin while that <50% is known as low methyl ester pectin (Liew et al., 2018). Pectin is broadly utilized both in the food (as gelling, thickening, and stabilizer agent) and in pharmaceutical (as bioactive components) industries including biomedical applications as inventive uses (Robledo & Vázquez, 2019). High‐methoxyl pectin (with DE >50%) fulfills a requirement to consider as a commercially available food‐grade pectin (Canteri et al., 2005). This high‐methoxyl pectin nature of banana peel pectin was reported by Rivadeneira et al. (2020) with DE 75.03%, which was extracted from unripe banana peels, and Khamsucharit et al. (2018) extracted the pectin (DE ranged: 63.15%–72.23%) from different varieties of banana peels. However, in the context of yield, the pectin extracted from banana peels was lower (9.75%–23.78%); similarly, papaya alone gives lower pectin as 12.9% (Altaf et al., 2015) more than the amount of pectin extracted from mixed peels of banana and papaya, which was reported by Maran and Prakash (2015) to be of 25.41%. This was the basic motivation for mixing (as 1:1) banana and papaya peels, in addition, to increasing the methoxyl content (MeO) of papaya peel pectin in the current study.

Pectin is additionally utilized in the preparation of an assortment of items including eatable and biodegradable films, adhesives, paper substitutes, foams and plasticizers, surface modifiers for clinical devices, materials for biomedical implantation, and drug conveyance (Roy et al., 2017; Sriamornsak, 2016). The complexity of the pectin structure gives structural epitopes that impart exceptional capacities. It has various constructive outcomes on human prosperity including bringing down cholesterol and serum glucose levels, reducing cancer, and stimulating the resistant response (Mohnen, 2018). In food industries, the physicochemical attributes of pectin decide the specificity of the use of pectin in distinctive foods.

## 2 MATERIALS AND METHODOLOGY

Both banana and papaya fruit peels were collected upon random sampling methods from different cafés, restaurants, and banana selling areas located in Arba Minch town, Gamo Zone, Southern Nations, Nationalities, and Peoples’ Region, Ethiopia. The collected fruit peels were washed separately with tap water to eliminate any sticky contaminations like dirt, mud, or dust particles. The peels were cut into small portions and blanched with boiling water at 95°C for 5 min to inactivate the enzyme. Furthermore, this solution was filtered over muslin fabrics. Insoluble materials (pieces) were treated with 97% ethanol for 30 min to remove the majority of ethanol‐soluble constituents. The residues were subjected to manual handpressing to eradicate leftover water. The obtained alcoholic‐insoluble solid was dried separately at 60ºC for one day using a hot air oven until achieving a constant weight. The dried‐out peel sample was ground well into a fine powder and preserved using a moisture‐proof bag, until it was used for the extraction of pectin (Koubala et al., 2014).

### 2.1 Experimental design

The response surface methodology (RSM) that includes the Box–Behnken design (BBD) and the central composite design (CCD) with the three‐level full factorial design to associate the correlation among process variables and responses (Jafari et al., 2017) was studied in common. Among them, BBD is more efficient than CCD and is also much more efficient than the three‐level full factorial designs (Ferreira et al., 2017).

Hence, the 3‐factor BBD was adopted in the present study to regulate the finest arrangement of variables for the extraction of pectin from mixed banana and papaya fruit peels. The Design Expert 11 statistical software was used for RSM determination. Furthermore, Design Expert 11 software was applied to minimize the experiments in the best technique. Here, in this study extraction temperature (*A*), pH (*B*), and time (*C*) were taken as the independent variables. The yield and anhydrouronic acid (AUA) % (Table 1) were nominated as the responses for the grouping of independent variables (Peng et al., 2020).

**TABLE 1:** Results of experimental runs with the actual and predicted values of dependent variables

Runs	Independent variables			Dependent variables			
				% Yield		AUA (%)	
	*A*: temperature (°C)	*B*: pH	*C*: Time (min)	Experiment results	Predicted results	Experiment results	Predicted results
1	90	2	25	21.08 ± 0.99^ab^	21.33	65.62 ± 1.11^ab^	65.68
2	70	3	25	21.68 ± 0.71^ab^	21.50	66.62 ± 1.74^ab^	66.71
3	70	2	35	23.78 ± 0.20^a^	23.22	66.63 ± 0.12^ab^	66.56
4	90	3	15	16.69 ± 0.38^d^	16.68	65.68 ± 0.77^ab^	65.56
5	90	3	35	21.06 ± 0.94^b^	21.12	66.12 ± 0.84^ab^	66.13
6	50	2	25	13.62 ± 0.56^e^	14.16	66.58 ± 1.48^ab^	66.55
7	50	3	15	9.75 ± 0.64^f^	9.44	66.62 ± 0.69^ab^	67.15
8	90	4	25	21.72 ± 0.79^ab^	21.17	64.50 ± 0.79^b^	64.43
9	70	3	25	21.53 ± 1.91^ab^	21.50	66.70 ± 1.05^ab^	66.71
10	70	3	25	21.73 ± 1.11^ab^	21.50	66.66 ± 2.03^ab^	66.71
11	70	4	15	17.04 ± 0.99 cd	17.59	66.38 ± 0.61^ab^	65.46
12	70	2	15	17.38 ± 0.01 cd	17.14	67.18 ± 0.23^a^	67.09
13	50	3	35	12.37 ± 1.23^ef^	12.38	65.85 ± 0.01^ab^	65.96
14	70	4	35	19.61 ± 0.73^bc^	19.85	65.87 ± 1.25^ab^	65.82
15	70	3	25	21.71 ± 1.32^ab^	21.50	66.63 ± 0.89^ab^	66.71
16	50	4	25	11.65 ± 0.92^ef^	12.40	65.52 ± 0.12^ab^	65.45
17	70	3	25	20.87 ± 0.84^b^	21.50	66.65 ± 1.03^ab^	66.71
*p*‐value				.000		.023	

Note

Experimental results described in mean ± SD (*n* = 3), which do not share the same letter of superscripts, indicate statistically significant; AUA: % anhydrouronic acid content.

### 2.2 Extraction and purification of pectin

The extraction of pectin was performed under microwave extraction, as described by (Rivadeneira et al., 2020) with slight modifications in the optimization of variables. Twenty grams (1:1 ratio of banana and papaya peels) of fruit peel powder was added into 200 ml of distilled water and made as a slurry in a 500‐ml conical flask. The pH of the slurry was attuned by using citric acid. The sample was dripping for 20 min at room temperature. The slurry was kept inside the microwave cavity and exposed by microwave energy at 100‐watt power for different time intervals and temperatures (Table 1). The microwave extracted samples were filtered using a muslin cloth and kept for further purification.

#### 2.2.1 Purification

Pectin‐containing aqueous extract was coagulated by adding twice the volume extract (2:1) of 97% ethyl alcohol (at 4°C) and left for 3 h. The obtained precipitated ethanol‐insoluble portion was purified by washing in 97% ethanol solution to remove unwanted pectin color and recovered through filtration. It was dried at 45°C in a hot air oven until attaining constant weight. Calculated the pectin yield as:

$$\%\;\text{yield}\;\text{of}\;\text{pectin}=\frac{A}{B_{i}}\times 100.$$

Where: *A* is the amount of extracted pectin (g) and *B*_i_ is the initial amount of fruit peel powder.

### 2.3 Physicochemical analysis of pectin

Physicochemical analyses of the optimized sample were characterized as follows.

1. Ash and moisture content was determined by AOAC (Association of Official Analytical Chemists) (2000).
2. Protein content was determined using the Kjeldahl nitrogen method.
3. Equivalent weight: It was determined by the process reported in Girma and Worku (2016). Equivalent weight was used for the determination of anhydrouronic acid (AUA) and the degree of esterification (DE).
4. Methoxyl content (MeO): It was performed by using the method described by Girma and Worku (2016).
5. Estimation of AUA: It was obtained by using the formula that was reported in the literature (Koubala et al., 2014) as described:

$$\text{AUA}\;\left(\%\right)=\frac{176\;(0.1\;z)\;100}{W\times 1000}+\frac{176\;(0.1\;y)\;100}{W\times 1000}.$$

Where: 176 g/mol – molecular weight of AUA; *z* – the volume of NaOH in equivalent weight, *y* – the volume of NaOH in methoxyl content, and *W* – sample weight, *g*.

- Degree of esterification: It was determined through MeO and AUA content (Daud et al., 2019). This was obtained as:

$$\text{DE}\;\left(\%\right)=\frac{176\times \%\;\text{MeO}\times 100}{31\times \%\;\text{AUA}}.$$

Where: % MeO – methoxyl content, % AUA – anhydrouronic acid content.

- Acetyl value was determined by the method reported in Virk and Sogi (2018).
- Functional properties of pectin powder: The water‐holding capacity (WHC), oil‐holding capacity (OHC), and swelling capacity (SC) were assessed by the method reported by Wongkaew et al. (2020).
- Solubility test of dry pectin in hot and cold water: The solubility test of pectin powder was determined by the process given in Kukwa et al. (2017).
- Solubility test of pectin in the organic solvent: Solubility of pectin in the organic solvent was determined by the method given by Tyagi and Yoges (2016).
- Solubility of dry pectin in cold and hot alkali was evaluated by the method described by Kukwa et al. (2017).
- FTIR spectroscopic study: Fourier‐transform infrared (FTIR) spectrum of the studied pectin was measured using PerkinElmer upon the frequency ranged 4000–400 cm^−1^ at a resolution of 4 cm^−1^ per second using the potassium bromide (KBr) pellet method.

## 3 RESULTS AND DISCUSSION

### 3.1 Pectin yield and anhydrouronic acid content

The experimental runs (17 runs were designed through the Box–Behnken design [BBD] method) were performed on the mixture of banana and papaya peels, and the obtained outcomes are described in Table 1. It illustrates that the percentage yield of pectin extracted (using microwave‐assisted extraction method) was found as ranging between 9.75% and 23.78%. These currently studied results are comparable with those of the earlier study (Maran & Prakash, 2015) which had resulted in the pectin (ranged: 7.62%–24.69%) from Carica papaya peel waste by using microwave‐assisted extraction. Upon the experimental circumstances, the responses’ % yield and anhydrouronic acid content (% AUA) of the studied experimental runs had to show statistical significance with the *p*‐values (for % yield: 0.000 and % AUA: 0.023). Among the experimental designs, run 3 (at temperature: 70°C, pH: 2, and time: 35 min) gives the maximum pectin yield (23.78%); whereas, run 7 (at temperature: 50°C, pH: 3, and time: 15 min) gives the minimum pectin yield (9.75%). Similarly, AUA % of experiments was found in the range of 64.50%–67.18%. This result of the study agrees with that of the earlier study (ranged 24.51%–67.98% of AUA content) of pectin which was extracted from cooking bananas (Diriisa et al., 2020). The current study resulted in the maximum AUA at run 12 (temperature: 70°C, pH: 2, and time: 15 min) which was 67.18%, however, the minimum AUA content was found in run 8 (at temperature: 90°C, pH: 4 upon 25 min) with the value of 64.50%.

#### 3.1.1 Influence of process variables on extraction yield

The experimental design and its corresponding results of the responses are described in Table 1. It is shown that a significant change in the yield of pectin was found. According to an earlier study (Xiaoxia et al., 2020), analysis (regression method) was performed on the experimental data and the predicted responses (yield of pectin and AUA content). It is obtained via the second‐order polynomial equation, which was used in the response surface method.

(1) $$Y=\beta 0+\sum_{i=1}^{k}\beta_{i}X_{i}+\sum_{i=1}^{k}\beta_{\mathrm{ii}}X1^{2}+\sum \sum_{i<j}\beta_{\mathrm{ij}}X_{i}X_{j}.$$

Where: *β_i_*, *β_ii_*, and *β_ij_* are regression coefficients, *X_i_* and *X_j_* are the coded independent variables, and *k* is the number of factors. The second‐order equation related to the % yield is:

(2) $$\begin{array}{cc} Y\;\text{(\textbackslash{}\% )}= & 21.50+4.24\;A‐0.7300\;B+2.09\;C+0.6525\;AB+0.6175\;AC‐0.9575\;BC\\ & ‐\;4.40\;A^{2}‐0.0908\;B^{2}‐1.96\;C^{2}. \end{array}$$

Where: *Y* – yield, *A* – temperature, *B* – pH, and *C* – time.

As seen in the equation, the coefficient with a greater value indicates that it has a greater impact on the response. Linear variables (*A* and *C*) and interaction terms (*AB* and *AC*) had a positive effect (means directly proportional) on the pectin yield due to positive coefficients (4.24, 2.09, 0.6525, and 0.6175). The positive effect of the coefficient reflects the additive effect. Whereas, linear variable (*B*), interaction term (*BC*), and quadratic variables (*A* ^2^, *B* ^2^, and *C* ^2^) indicated negative or antagonistic effects against pectin yield.

Analysis of variance (ANOVA) was used for examining the obtained results (Table 2 **)**. It shows that the *F*‐value (104.93) and the allied *p*‐value (*p* < .0001) designate that the presently used regression model is found as significant. It seems that there is only a 0.01% chance that occurs due to noise. Linear variables (*A*, *B*, and *C*) within the group have a significant difference with lower *p*‐values (*p* < .05), and the interaction terms (*B*–*C*) among the group have a significant difference with a lower *p*‐value (.0108). The lack‐of‐fit was found as insignificant (with *F*‐value: 4.14) and the pure error was low, due to the high precision of the experimental yield as shown in Table 2. The lack‐of‐fit test indicates that independent variables have considered the effect on the response if it is valued as insignificant. The betterment of fit of the model was tested by the determination coefficient (*R* ^2^), adjusted coefficient (adj‐*R* ^2^), predicted coefficient (pre‐*R* ^2^), coefficient of variance (CV), and signal‐to‐noise ratio (S/N). The high value of *R* ^2^: .9926 and adj‐*R* ^2^: .9832 of this current study indicated that the model represents that the actual relationship is found to be well correlated among the response and independent variables. Furthermore, a low value (3.01) of the coefficient of variance (CV) supports great precision and worthy reliability of the experimental values. There is only a 10.18% (shown by pre‐*R* ^2^: .9082) chance that the lack‐of‐fit *F‐*value could occur due to noise. The predicted *R* ^2^ value (.9082) was found to be in good agreement with the adj‐*R* ^2^: .9832, with the difference being about < 0.2%. In the present study, the adequate precision was found to be 32.3 which indicates the best fitness of the developed model.

**TABLE 2:** ANOVA results for extraction % yield from mixed fruit wastes

Source	Sum of squares	DF	Mean square	*F*‐value	*p*‐value
Model	291.90	9	32.43	104.93	< .0001
A	143.48	1	143.48	464.20	< .0001
B	4.26	1	4.26	13.79	.0075
C	34.78	1	34.78	112.51	< .0001
AB	3.70	1	3.70	12.51	.0213
AC	1.53	1	1.53	4.93	.0618
BC	3.67	1	3.67	11.86	.0108
A^2^	81.36	1	81.36	263.21	< .0001
B^2^	0.347	1	0.347	0.1122	.7475
C^2^	16.19	1	16.19	52.37	.0002
Residual	2.16	7	0.3091		
Lack of fit	1.64	3	0.5455	4.14	.1018
Pure error	0.5271	4	0.1318		
Total	294.06	16			

Fit statistics of % yield:	
SD = 0.5560	Adjusted *R* ^2^ = .9832
Mean = 18.47	Predicted *R* ^2^ = .9082
CV (%) = 3.01	Adeq precision = 32.3167
*R* ^2^ =.9926	

Abbreviations: CV, coefficient of variance; DF, degree of freedom; R^2^, regression coefficient.

#### 3.1.2 Influence of process variables on the anhydrouronic acid content

Response surface regression model for the AUA content of pectin is represented by Equation (3) as:

(3) $$\begin{array}{cc} \text{AUA}\;(\%)= & 66.27+0.3487\;A‐0.4675\;B‐0.1512\;C+0.6852\;AB‐0.4350\;AC\\ & ‐\;0.1675\;BC‐0.7380\;A^{2}‐0.3255\;B^{2}+0.2220\;C^{2}. \end{array}$$

The AUA content of pectin was analyzed by ANOVA, as shown in (Table 3). The studied results obtained as high *F*‐value (61.31) with a low *p* **‐**value (*p* < .0001) do indicate that the regression model becomes significant; the *p*‐value makes it evident that there is only a 0.01% chance that nonsignificance could occur due to noise. The betterment of fit of the model was confirmed by the higher coefficient (*R* ^2^: .9875), adjusted coefficient (adj. *R* ^2^: .9714) with a lower coefficient of variance (CV: 0.1670), and signal‐to‐noise ratio (shown in Table 3).

**TABLE 3:** Analysis of variance (ANOVA) results for anhydrouronic acid (AUA) content (in %) of pectin

Source	Sum of squares	DF	Mean square	*F*‐value	*p*‐value
Model	6.77	9	0.7515	61.31	< .0001
A	0.9730	1	0.9730	79.34	< .0001
B	1.75	1	1.75	142.57	< .0001
C	0.1830	1	0.1830	14.92	.0062
AB	0.1272	1	0.1272	12.22	.0098
AC	0.7569	1	0.7569	61.72	.0001
BC	0.1122	1	0.1122	9.15	.0192
A^2^	2.29	1	2.29	187.00	< .0001
B^2^	0.4461	1	0.4461	36.38	.0005
C^2^	0.2075	1	0.2075	16.92	< .0045
Residual	0.0858	7	0.0123		
Lack of fit	0.0547	3	0.0182	2.34	.2142
Pure error	0.0311	4	0.0078		
Total	6.85	16			

Fit statistics of anhydrouronic acid (%):	
SD = 0.1087	Adjusted *R* ^2^ = .9714
Mean = 66.33	Predicted *R* ^2^ = .8677
CV (%) = 0.1639	Adeq precision = 25.6413
*R* ^2^ = .9875	

Abbreviations: CV, coefficient of variance; DF, degree of freedom; *R* ^2^, regression coefficient.

Lack of fit of the studied model was found to be higher than 0.05 (insignificant); also, *F*‐value for the lack of fit found to be 2.34 (Table 3) for these parameters designated that the mathematic model in Equation (3) is well adapted to the response for expecting AUA content of pectin with the variables. There is a 21.42% (with the *p*‐value: .2142) probability that lack‐of‐fit with the *F*‐value: 2.34 could occur due to noise. The predicted *R* ^2^ (.8651) is in reasonable agreement with the adjusted *R* ^2^ of .9714 (i.e., the difference is <0.2). Adequate precision measures the signal‐to‐noise ratio and the ratio >4 is desirable. Thus, in this study, the adequate precision was found to be 26.0936, which designates the greatest suitability of the established model.

**FIGURE 1:**
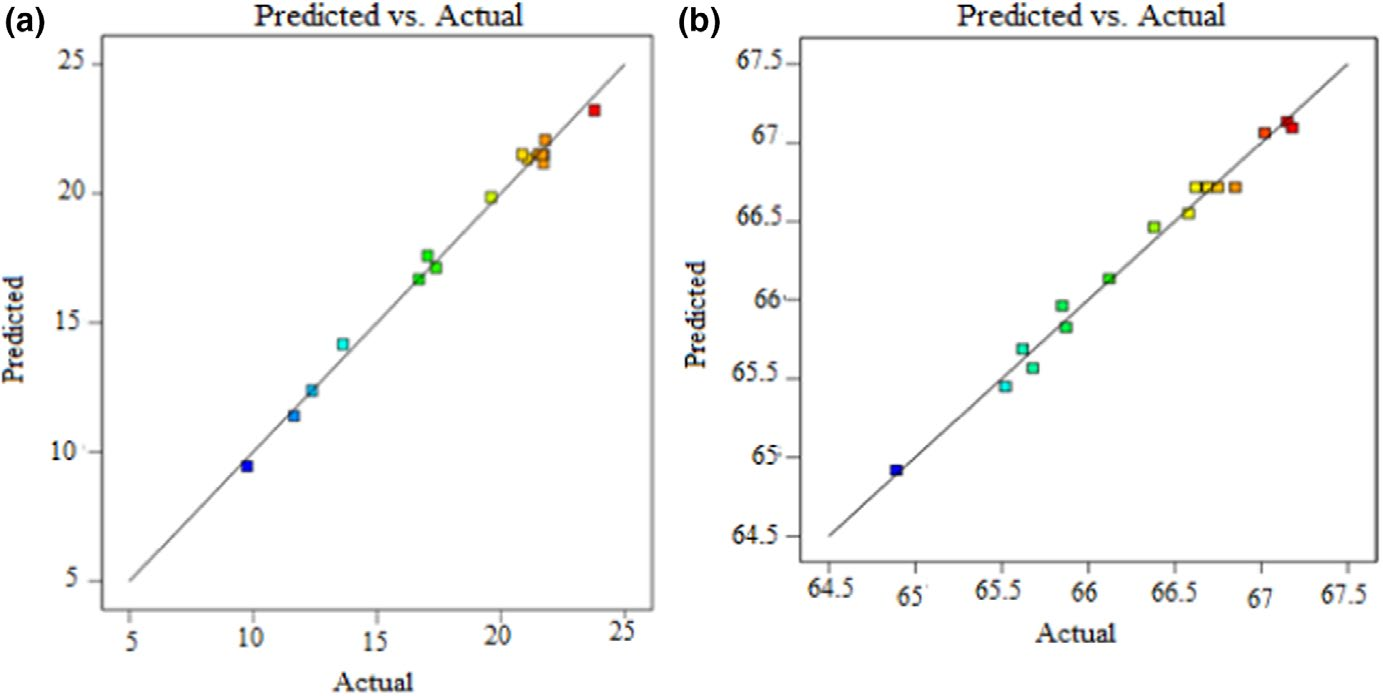
Predicted vs. actual % yield (a) and predicted versus actual % AUA (b)

**FIGURE 2:**
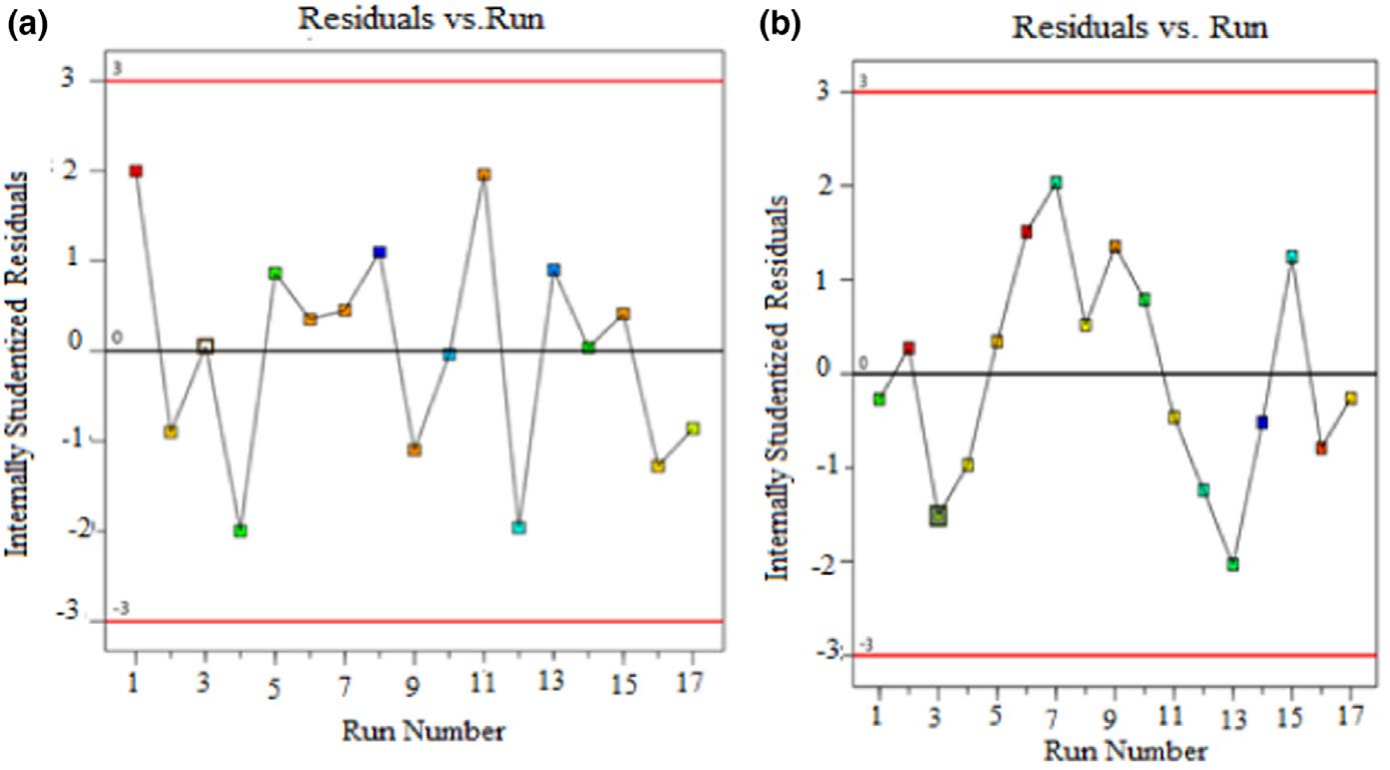
Diagnostic plots for developed model adequacy: (a) Yield (%) and (b) AUA (%)

The predicted and actual results of this study (shown in Figure 1) were compared, and it is found to be adjacently dispersed in both cases (% yield and AUA %). This designates the least deviation and closest agreement among the values of predicted and actual. Furthermore, as seen in Figure 2, all the obtained results that were placed within the permissible limits (−2 to +2) were selected; it indicates that the developed model has an adequate fit for the experiments.

### 3.2 Single‐factor effect on the extraction of pectin

#### 3.2.1 Effect of temperature

**FIGURE 3:**
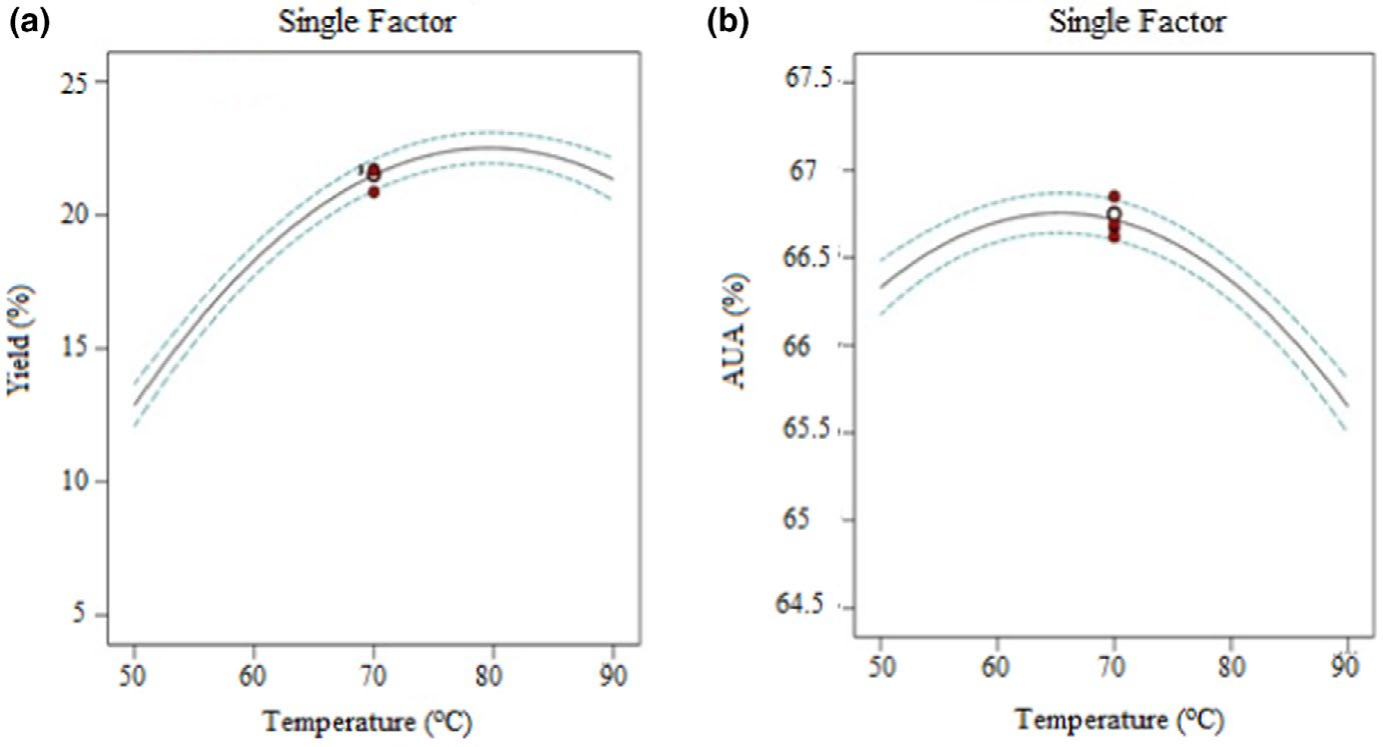
Single‐factor effect plot on the extraction of pectin from mixed banana‐papaya peels: (a) pectin yield vs. temperature; (b) anhydrouronic acid vs. temperature

Temperature is the factor that commonly affects the amount of extraction yield and purity (in % AUA) of pectin. The results shown in Figure 3 indicate that the pectin yield was found to be increased up to some extent; later decreased upon raising the temperature during the extraction of pectin. This is due to an increase in temperature that could disturb the hydrogen bonds and ester; then improve the solubility and solvent diffusion to the plant tissues and increase the extraction yield of pectin. However, further increment of temperature shows the reduction of pectin yield. It might be that higher temperature leads to the breaking of pectin molecules and improves the de‐esterification of polygalacturonic chains; also, the dark‐brown color of pectin powder loses the purity of the product (Guo et al., 2016).

#### 3.2.2 Effect of pH

The pH means that at low pH, the amount of pectin yield and the purity of extracted pectin were found to be higher. This observation revealed that there was a good agreement with the earlier study (Zakaria, et al., 2021), with the highest pectin yield obtained at pH 2.0. This may be due to the decrease in pH when the number of hydrogen ions increases, resulting in the neutralization of more carboxylic groups of pectin which does make higher pectin yield.

#### 3.2.3 Effect of time

**FIGURE 4:**
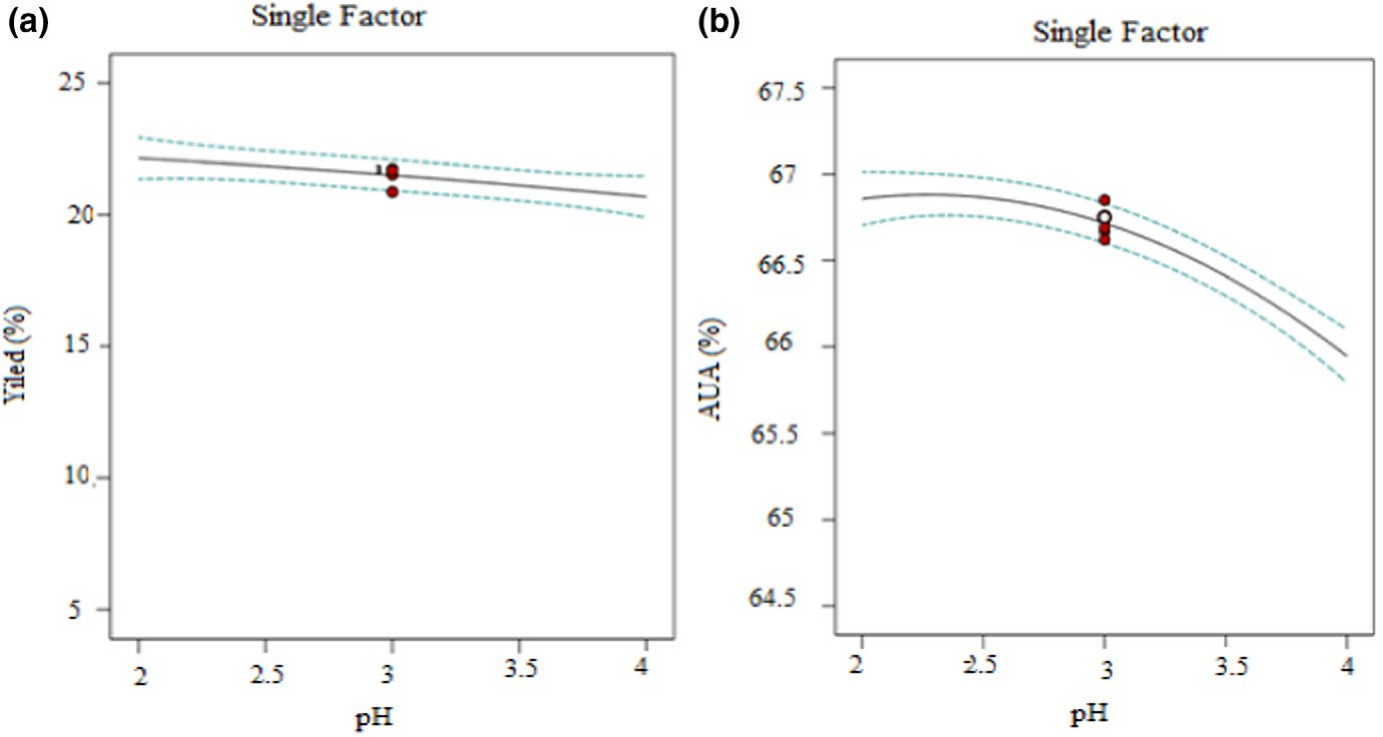
Single‐factor effect plot on the extraction of pectin from mixed banana‐papaya peels: (a) pectin yield vs. pH; (b) anhydrouronic acid content vs. pH

**FIGURE 5:**
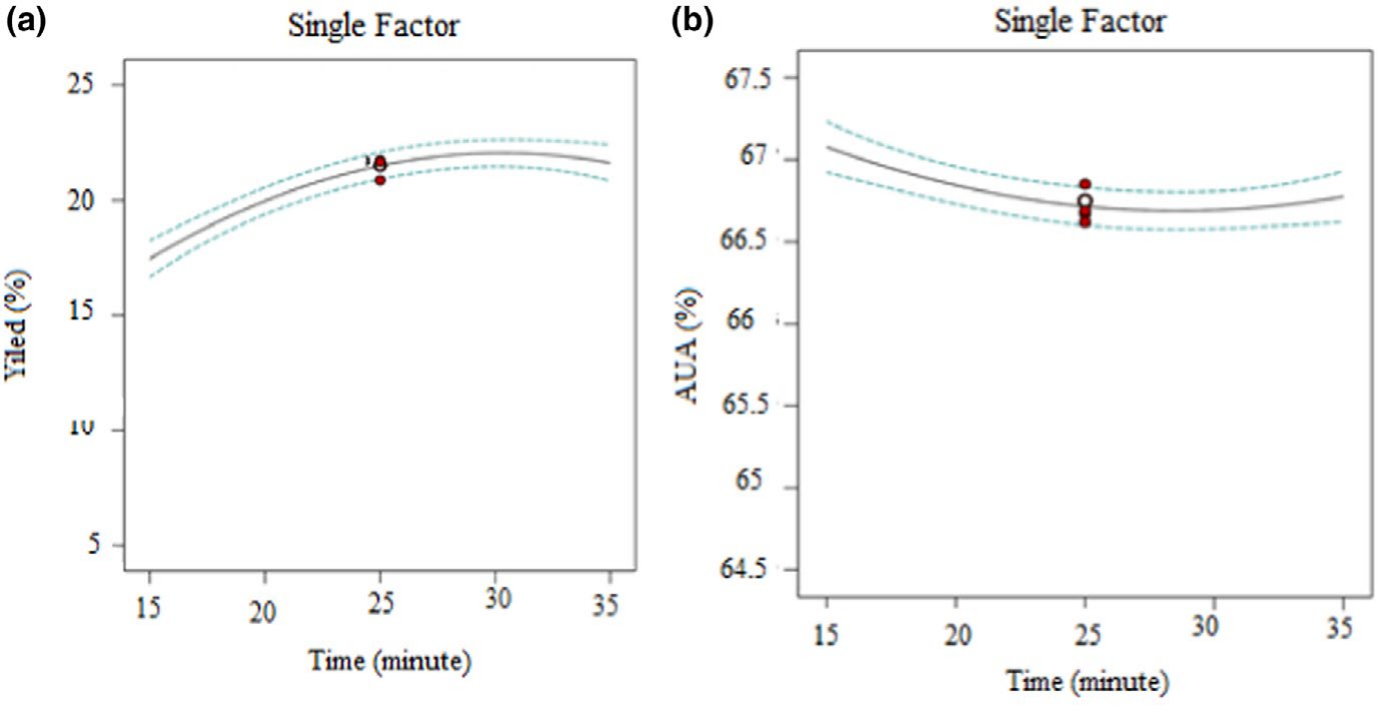
Single‐factor effect plot on the extraction of pectin from mixed banana‐papaya peels: (a) pectin yield vs. time; (b) anhydrouronic acid content vs. time

Figures 4 and 5a shows that the effect of time on pectin extraction demonstrated that an increase in the response (% yield) takes place. This enhancement of extraction revealed in the current study might be due to the extraction under the microwave‐assisted method, which has taken a shorter time than the other methods of extraction reported (Rivadeneira et al., 2020). Pectin yield was found to be lower at the beginning of extraction, and there may still be some pectin attached to the cell wall. Generally, the color of the pectin became dark‐brown for lengthier times of extraction indicating that the extract might have required a higher amount of alcohol for washing the precipitate, which leads to low‐quality pectin.

### 3.3 Influence of interactive parameters among variables

#### 3.3.1 Influence of temperature and pH

**FIGURE 6:**
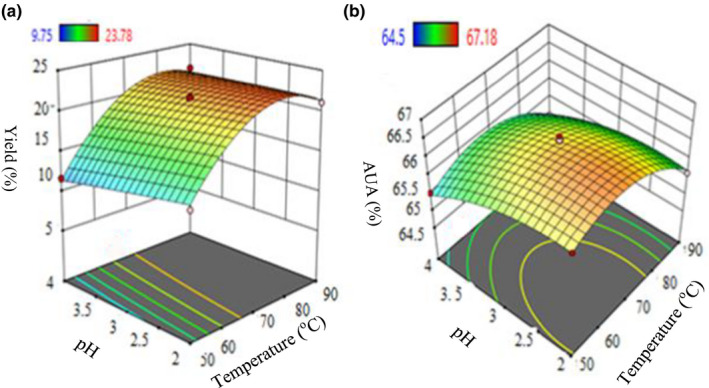
3D response surface interaction plots for the effect of temperature and pH on % yield (a) and % AUA (b)

Extraction of pectin in different process variables (data presented in Table 1) was visualized through a three‐dimensional (3D) response surface plot. The surface plots in figures were drawn between any two variables by keeping the one variable constant. The surface plot (Figure 6a) displays at a constant extraction time (25 min); the increases of temperature (up to 70°C) indicate an upturn in the percentage yield up to some extent (23.78%) and decreased then beyond 70°C. This could be upon increasing the temperature that leads to the increment of solubility that influenced on enhancing the rate of extraction as similar to an earlier report (Jin et al., 2019).

Furthermore, the plant cell tissues may be destroyed at elevated temperatures, then the diffusion of the solvent into the cell walls might be speeded up and an additional amount of pectin gets extracted and dissolved into the medium which leads to the notable increases in the extract. But, to some extent high temperatures lead to thermal degradation and result in a lower amount of pectin (Girma & Worku, 2016). In the present study, the results were found as the maximum pectin yield of 23.78% at 70°C; and during later extension of temperature (exceeding 70°C), the yield of pectin could get decreased, as supported by an earlier study which was done by Girma and Worku (2016) when the temperature exceeded 70°C. As seen in (Figure 6a), increasing the pH at a constant treatment time (25 min) leads to decreasing percent yield. The highest amount of pectin (23.78%) was obtained at pH 2.0; this might be a lower pH, which raises the H^+^ concentration in the medium that stimulates the hydrolysis of protopectin and repressed the highly hydrated carboxylate groups; and therefore, it is transformed into hydrated carboxylic acid groups which lead to a higher pectin yield. This is in good agreement with Shan et al. (2014); Udonne et al. (2016) who had studied the pectin extracted from passion fruit peel, apple‐pomace, and sugar‐beet pulp, which was reported as increases in the pectin amount upon the increment of acid strength.

Similarly, experimental run: 12 (temperature: 70°C & pH: 2) shows the highest AUA % (67.18%); then with further rises of temperature, the AUA % gets reduced (Table 1 and Figure 6b). This might be due to occurrence of the side reactions on galactose (Gal) residues under β‐elimination and oxidation at high temperatures (Xiaoxia et al., 2020). The steepness of response surface plots (Figure 6) was observed in response to % yield and AUA %, which showed that temperature had a great influence.

#### 3.3.2 Effect of temperature and time

**FIGURE 7:**
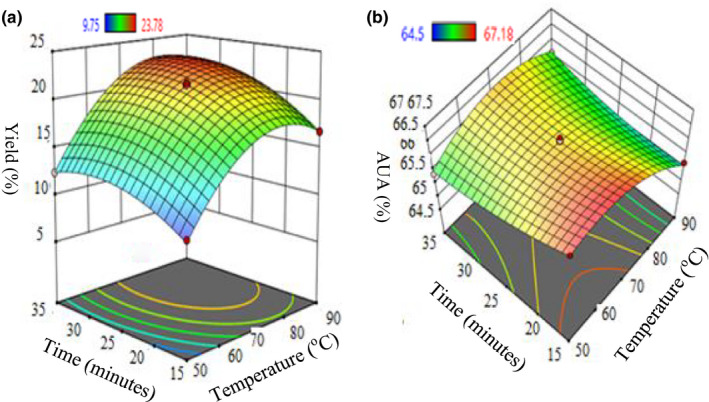
3D response surface interaction plots for the effect of temperature and time on extraction % yield (a) and % AUA (b)

In Figures 7a,b, the 3D surface plot (at constant pH: 3) shows that an increase in time and temperature leads to a gradual increment of the percentage yield. It is revealed that elevated period of extraction supports the retrieval of pectin. A similar trend of results was obtained in an earlier study (Sijin et al., 2015); this indicated that more time is required for maximum elution of pectin into the solvent where the liquid had to enter the dry peel powder leading to the dissolution of pectin, and successively eluted it from the plant‐cell wall (Pawadee et al., 2018). Figures 7b 3D plot shows that at a constant pH value (B: 3), a rise in time leads to a decrement of AUA %. Maximum AUA % at constant pH: 3 was obtained as 67.18 within 15 min of extraction under microwave‐assisted approach.

#### 3.3.3 Effect of pH and time

**FIGURE 8:**
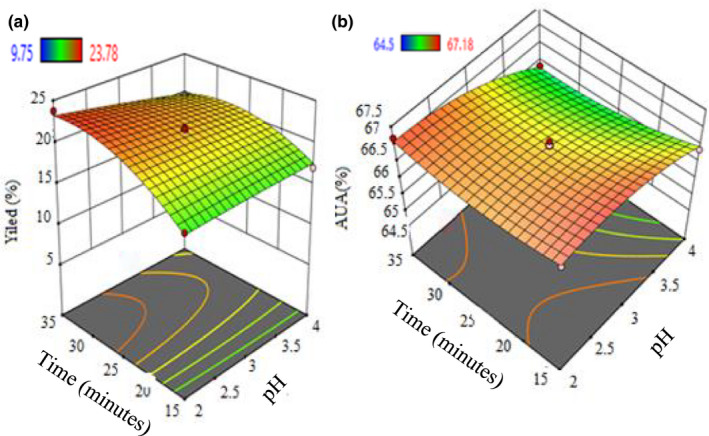
3D response surface interaction plots for the effect of time and pH on % yield (a) and % AUA (b)

Three‐dimensional (3D) response surface plots (Figure 8a,b) were plotted by the interaction between time and pH at constant temperature 70°C. Figures 4 and 8a,b makes it evident that the extraction time of 35 and 15 min contributed a maximum pectin yield (23.78%) and AUA % (67.18), respectively. It shows that the higher time of extraction leads to a linear enhancement in percentage yield. This resulted in the current study being in a good agreement (but achieved more yield of pectin within the shortest extraction time) with Shan et al. (2014) who had extracted the pectin (12.3% yields) from the passion fruit peel waste under thermal extraction. The yield of pectin increases with a longer period of extraction, which was because greater time provides more extraction opportunities. Thus, in the present study a higher amount (23.78%) of pectin got extracted within 35 min; this is because of the utilization of microwave heating for pectin extraction.

Figure 8a,b were obtained upon the interaction between time and pH at constant temperature 70°C. Figures 4 and 8a,b make it evident that the extraction time of 35 and 15 min contributed a maximum pectin yield 23.78% and AUA %: 67.18, respectively. It shows a linear increase in percentage yield upon enhancing the time. This resulted in the current study being in a good agreement (but achieved more yield of pectin within the shortest extraction time) with Shan et al. (2014) who had extracted the pectin (12.3% yields) from passion fruit peel waste under thermal extraction. The yield of pectin increases with a longer period of extraction; this is due to the longer time offering more opportunities to extract the pectin. Thus, in the present study a higher amount (23.78%) of pectin got extracted within 35 min; this is because of the utilization of microwave heating for pectin extraction.

The pH is the vital factor that impacts the amount of extracted pectin (shown in Table 1 and Figures 6 and 8). This has also proved that while pH interacts with other parameters, time and temperature display the significant difference as *p*‐values .01 and .05, respectively (Table 2). Especially, the interaction of pH with time has shown to be well statistically significant. Hence, the pectin yield and AUA % are greatly influenced by pH as decreases while increasing the pH that the greater amount of pectin (23.78%) and AUA % (67.18) was reported at pH 2.0. It confirms that at low pH (greater H^+^) that helps to extract the higher amount of precipitated pectin. Furthermore, an upturn in the pectin yield is also noticed by the decreasing size of the powdered peels. This happens due to the availability of surface area that helps to increase the mass transfer through decreasing the size, thus increasing the pectin yield (Alok et al., 2017).

### 3.4 Validation of optimized conditions

The numerical optimization method was used in the present study that used to optimize the variables for % yield of pectin and AUA % (as responses) by employing with Design‐Expert software version 11 with the maximum desirability values.

**FIGURE 9:**
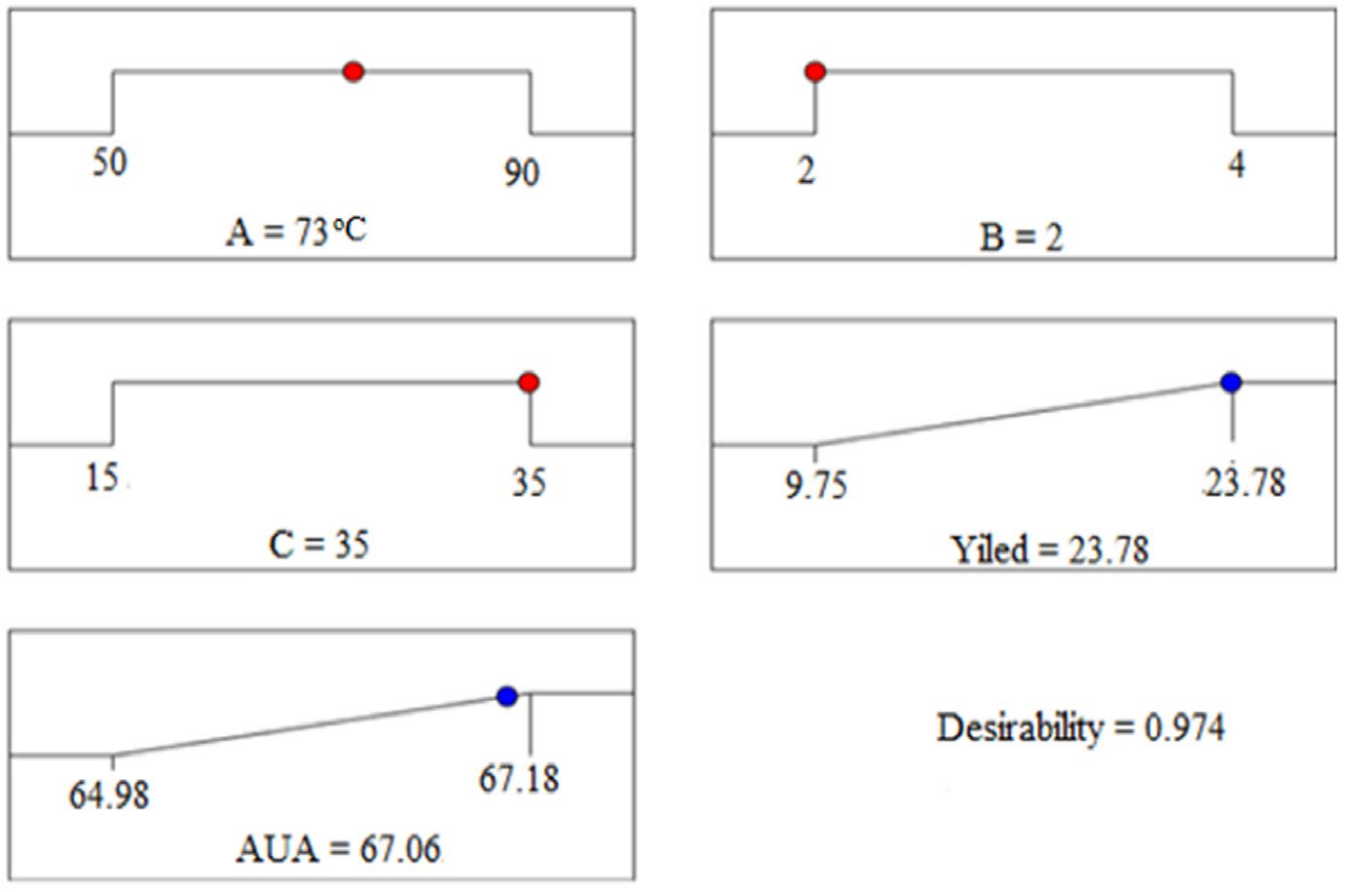
Ramp desirability plot optimization of the variables (*A*: temperature, *B*: pH, and *C*: time) for the best of pectin extraction represent in % yield and AUA content Note: *A*: Temperature; *B*: pH; *C*: Extraction time; AUA: Anhydroronic acid content (in %)

To verify the stability and accuracy of the experiments; based on higher desirability values better, three optimum experimental runs were selected as with their corresponding optimal conditions (shown in Table 4). Among these three better experimental runs, the best‐optimized condition (with the higher desirability value: 0.974) was selected as found to be temperature 73°C, pH: 2, and time 35 min with the higher yield of pectin and AUA as 23.78% and 67.06%, respectively (Figure 9). This best‐optimized condition was verified through the estimation of % yield and % AUA of the mixed banana–papaya peel pectin extract. These results found (% yield: 23.74 ± 0.25 and AUA: 67.27 ± 0.05%) were nearly equal to the response predicted from the software (i.e., 23.78% pectin yield and 67.06% AUA), which confirms that response surface methodology (RSM) might be used effectually for optimizing the factors in complex processes. This optimized temperature of the present study allied with the optimum temperature was reported (Chandrasekaran et al., 2017) with different optimum times and pH values with a predicted value of 3.98% pectin yield. This might be dependent on the extraction method; here in the present study the higher pectin was extracted by adopting microwave extraction, which enhances the pectin extraction from the mixed peel (banana and papaya) wastes.

**TABLE 4:** Best of three optimized conditions for the extraction of pectin from mixed banana and papaya peels

Temperature (°C)	pH	Time (min)	Yield (%)	AUA (%)	Desirability	
73.0	2.000	35	23.78	67.06	0.974	Selected
73.3	2.000	35	23.80	66.86	0.972	
73.5	2.000	35	23.83	66.86	0.970	

Note

AUA: % anhydrouronic acid content.

### 3.5 Physicochemical assessment of extracted pectin

The physicochemical analyses of pectin (optimized) obtained from mixed papaya and banana peels under the optimized conditions were subjected to appraise the purity and fitness in milk products. The obtained results of physicochemical attributes are presented in Table 5.

**TABLE 5:** Physicochemical characterization of the extracted mixed banana–papaya pectin

Characteristics	Results obtained	IPPA standard	Literature values	Reference
Equivalent weight (g/ml)	783.69 ± 0.46	600–800	326.7–1428.57	Wonago (2016)
Methoxyl content (%)	8.37 ± 0.42	2.2–7.8	8.89	Girma and Worku (2016)
Anhydrouronic acid (%)	69.97 ± 0.05	min. 35	63.71–77.41	Wonago (2016)
Degree of esterification (%)	67.91 ± 0.33	‐	61.19–70.79	Roy et al. (2017)
Moisture content (%)	7.2 ± 0.27	max. 12	8.33	Kukwa et al. (2017)
Ash content (%)	6.20 ± 1.26	max. 10	5.7	Mohamed and Mohamed (2015)
Protein content (%)	3.92 ± 0.05	–	3.8–7.6	Guo et al. (2016)
Acetyl value (%)	0.48 ± 0.11	–	0.43 ± 0.01	Ramidan (2015)
pH	4.87 ± 0.04	–	4.56 ± 0.05	Tyagi and Yoges (2016)

Note

Data are expressed as mean ± standard deviation (*n* = 3).

#### 3.5.1 Equivalent weight

It is the total content of free galacturonic acid (not esterified) in pectin (Altaf et al., 2015). Also, it depends on the pH and solvent used for extraction and the number of free acids available on pectin (Muthukumaran et al., 2017). The equivalent weight (EW) of pectin is a sign of gel‐forming capacity. If the equivalent weight may be greater, it reflects to achieve a higher gel‐forming ability (Wongkaew et al., 2020). The EW of the presently studied mixed banana–papaya peel pectin under optimized conditions was found to be 783.69 ± 0.46. The result shows that the pectin obtained under microwave extraction (MAE) has a higher equivalent weight (783.69 ± 0.46) as compared to that pectin extracted from papaya (*Carica papaya* Linn.) (EW: 455), which was used in hot acid extraction (Altaf et al., 2015). But lower values of EW than that of pectin were extracted from banana (925.01) and mango peel (895.00) reported by Girma and Worku (2016). The lower value of EW was obtained in the present study, which could be due to pectin polymerization at lower pH, method of extraction, and maybe the nature of fruit peels used for extraction.

#### 3.5.2 Methoxyl value

Methoxyl (Mox) value represents the pectin distribution capacity in water; gel capacity of high Mox may suggest strong cohesive and adhesive forces which might infer with the increment of firmness of the food products (Mugampoza et al., 2020). The Mox value of the studied pectin (obtained at temperature: 73°C, pH: 2, and time: 35 min) was found to be 8.37 ± 0.42, which was relatively higher than the methoxyl content of standard pectin that was reported in the International Pectin Producers Association (IPPA, 2014) that ranged from 2.5 to 7.8. Commonly, the Mox of pectin is to be affected by the type of sample, raw materials’ quality, extraction methods, and titration process (Muthukumaran et al., 2017; Rury et al., 2017). The results of the current study were supported by earlier reports (Girma & Worku, 2016) which reported the Mox value (as 8.89) of mango peel pectin; but lower than the Mox value of pectin (19.33 ± 0.04), which was also extracted from mango peel under microwave‐assisted extraction (Wongkaew et al., 2020). This variation designates that the extraction method does play an important role in the extraction of any biological compounds from plant cells.

#### 3.5.3 Degree of esterification

The degree of esterification (DE) is the principal property for pectin application in the food industry, as it controls the gel‐forming effect of pectin (Daud et al., 2019). The DE of pectin obtained in the present study was found as 67.91 ± 0.33 (Table 5), which was found in the range: 63.29 ± 0.84–75 ± 0.53% of DE was reported from orange peels pectin extracted using citric acid (Sayah et al., 2016). Also, the current data are well aligning with the DE (61.19%–70.79%) of pectin from pomelo peel at pH: 2, which was reported earlier (Roy et al., 2017). But, the studied DE of the pectin is found to be lower than other studies, as the DE (75.03%) of pectin was extracted from unripe banana peels (Rivadeneira et al., 2020). Hence, the DE of pectin can be different depending upon ripeness, a portion of the fruit, origin, and separation methods (Altaf et al., 2015).

The results of DE obtained, which show that the pectin extracted at optimized conditions in this study might be classified as high‐methoxyl pectin with DE >50%, fulfill a requirement to consider as commercial food‐grade high‐methoxyl pectin (Canteri et al., 2005). The studied pectin shows the high‐methoxyl natured pectin of the mixed banana and papaya peel pectin (DE: 67.91 ± 0.33%), which is found to be in good agreement with the banana peel pectin reported by Rivadeneira et al. (2020) (with DE: 75.03%) extracted from unripe banana peels, and Khamsucharit et al. (2018) extracted the pectin from different varieties of banana peels (with DE: 63.15%–72.23%). Also, high‐methoxyl pectin is in the form of pectin traditionally used for canning applications. It is very sensitive to acidity (Pinheiro, 2008). DE (67.91 ± 0.33) of the presently studied pectin is comparatively lower than that of pectin from citrus fruit (Pinheiro, 2008). In this context, the studied pectin may be considered as high‐methoxyl pectin which has been used in the food industry as a gelling agent, thickening agent, and stabilizer. Furthermore, the studied pectin can also be used as a fat substitute in baked goods and to stabilize acidic protein drinks such as drinking yogurt that is supported by the reported literature (Marcon, 2005).

#### 3.5.4 Anhydrouronic acid content

The anhydrouronic acid (AUA) content in the studied pectin was found to be 69.97 ± 0.05. This is relatively comparable to that previously reported for the Kluai Nam Wa banana peel pectin with an AUA content of 69.67 ± 0.02 (Khamsucharit et al., 2018). The obtained AUA % has also been found to be eligible to meet the quality standard of pectin based on the IPPA (2014, 2014), with the minimum standard being 35%. The anhydrouronic acid could depend, based on raw materials, solvent, and extraction methods (Rury et al., 2017). Based on the AUA content on the presently studied mixed banana–papaya peel pectin obtained at optimized variables, the criteria for the use of commercial pectin were met. From this point of view, mixed banana/papaya peel wastes are considered as similar to other and potential pectin sources as like apple (*Malus Pumila. Cv Amri*) peel waste that has 62.82 ± 0.12% of AUA (Virk & Sogi, 2018) and orange peel (that has 93.28% of AUA) were reported (Devi et al., 2014). A minimum value (65% of AUA) was recommended by FAO to use as commercial pectin. Hence, the studied pectin does fulfill the requirement of FAO standard, and it is said to be an alternative potential pectin to use for industrial purposes, mainly to use as a stabilizing agent for yogurt‐like semisolid and liquid food products in food industries.

#### 3.5.5 Ash content

Ash content represents the available minerals like potassium, sodium, magnesium, and iron. The mineral matters in pectin are designated by the ash content. It also states the purity level of pectin, as per which higher purity pectin is designated by the low ash content (Castillo‐Israel et al., 2015). Additionally, the amount of ash is influenced by the presence of inorganic matters contained in fruit peels and the method of extractions (Sarah et al., 2018). Ash content found in this study under optimum conditions was about 6.20 ± 1.12%, which is lower than the IPPA standard (10%). Many researchers showed different ash contents of fruit peel pectins such as as cocoa peel that has 1%–5% (Sarah et al., 2018), orange peel that has 5.6%–9.7% ash (Tyagi & Yoges, 2016), 3.1% and 2.45%–6.66% of ash in orange peel using citric and nitric acid, respectively (Amice, 2016; Devi et al., 2014), and 7.2% and 4.8% of pectin using papaya (*Carica papaya* Linn.) peel on treatment with hydrochloric acid and citric acid, respectively (Altaf et al., 2015). The marked difference between the literature values and the current study depends on the method or purification process carried out and the nature of the fruit peels used for extraction. Also, as the food‐additive pectin must fulfill the standards of purity and quality. Hence, the pectin obtained in the present study is found to be in good agreement with earlier researches, and it is to be considered as good quality pectin.

Generally, commercial pectin does recommend with lower ash content (in terms of purity concern) as <10%. Hence, since lower ash content is more advantageous for gel formation, it might be reduced by washing with acidified alcohol or other processing methods (Begum et al., 2014). Consequently, the use of pectin with the desirable ash content depends on the application.

#### 3.5.6 Moisture content

Generally, the availability of moisture of any products (including pectin) does affect the shelf life; greater water content is the source that is susceptible to microbial activity and diminishes the value of pectin (Sarah et al., 2018). According to the quality standards of commercial pectin or IPPA standard (maximum acceptable limit of moisture: 12%); so, all kinds of pectin might not be higher than 12%. Pectin has a low moisture content that helps to inhibit the growth of microorganisms with better storage; but it may affect the pectin quality due to the production of pectinase enzymes (Muhamadzadeh et al., 2010).

The moisture of the presently extracted pectin was found as 7.2%, which is in the acceptable range (<12%) to store in a safe place. Its comparable and closer agreement with the values of pectin extracted (7.3%) from papaya (*Carica papaya* Linn.) peel by using citric acid as the chelating agent has been reported (Altaf et al., 2015). Also, the result of the current study supported by another study has concluded that the available moisture in pectin from different citrus peels lies in the range of 6.4%–10% (Khan et al., 2017). This might be due to the alteration in the hygroscopic nature of pectin with different DEs (Mohamed & Mohamed, 2015).

#### 3.5.7 Acetyl value

The acetyl value of the optimized pectin powder used in this study was reported as 0.48 ± 0.11. It is lower than those of the water‐soluble pectin extracted from red‐ and white‐ type grapefruit peels (AV: 0.55 and 0.52) and acid‐soluble pectin (AV: 0.61 and 0.60) was reported (Mohamed & Mohamed, 2015), and pectin extracted from apple (has AV: 0.68 ± 0.04) reported earlier (Khamsucharit et al., 2018). Acetyl value of this study extracted pectin from mixed banana and papaya peel wastes at optimum condition gives good gelling power with a lower acetyl value (0.48 ± 0.11), which was found to be lower than the pectin extracted from grapefruit peels and apple peel that was reported (Khamsucharit et al., 2018; Mohamed & Mohamed, 2015).

#### 3.5.8 Protein content

The amount of protein from the studied pectin extracted from mixed banana and papaya peel was found to be 3.92 ± 0.054%. It is in the range of 3.8%–7.6% protein obtained from sugar‐beet pectin (Guo et al., 2016). But, the pectin obtained in the present study is lower than the protein (8.6%–10.3%) in water‐soluble pectin from sugar beet (*Beta vulgaris* L.), which was reported recently (Lara et al., 2021). The earlier study (Li et al., 2015) describes the cause of the covalent linkages among pectin and protein, which might be the extraction medium (weak or strong acids) and precipitation method as well as the concentration ethanol at which pectin could be precipitated. Also, the protein availability is strongly influenced by the higher temperature, extraction time, and lowered pH; so forceful situations may contribute to the hydrolysis of protein. Since the proteinaceous component connected to the polymer chains of pectin has been evidenced to show a direct role in activating and stabilizing properties of pectin powder. The quantity of protein in pectin is a vital component for various food applications (in an emulsion). The protein behaves as the medium among the pectin and the produced oil droplets in an emulsion, resulting in a persistent system (Rivadeneira et al., 2020).

#### 3.5.9 Measurement of pH

The pH is the most essential parameter that determines microbial propagation and this parameter plays a vital role in assuring the product's suitability for preservation (Bruno et al., 2020). The pH of the currently resulting product (pectin) was reported at 4.02 ± 0.04. Comparably, this value was found as faintly lower than the pH of the studied extracted pectin from banana (4.60), orange (4.30), and lime (4.15) (Omoniyi et al., 2019). Since the pH values may be lower than 4.5; the control of pathogenic bacteria development is preferred throughout storage (Bruno et al., 2020). This is confirmed that where the pectin gets extracted from the banana peels mixed with papaya peels that control the growth of bacteria (with lower pH: 4.02 in the current study) during the storage rather than pectin extracted alone from the banana peel (shown higher pH: 4.60). The variation in the pH of pectin solution mostly deepened on the acid used for pH adjustment, type of fruit used for extraction (alone or mixed), and purity of ethanol used for the washing of pectin after alcohol precipitation.

### 3.6 FTIR analysis of pectin

**FIGURE 10:**
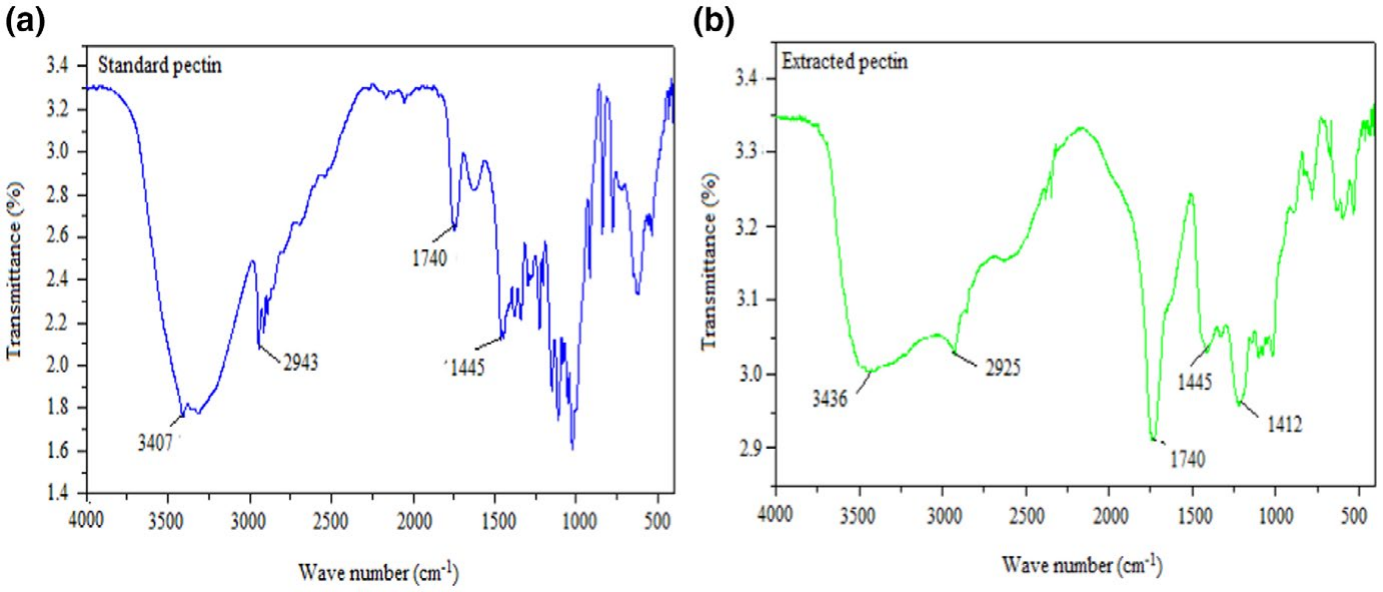
FTIR spectrum of standard (a), and pectin extracted (b) from the mixed banana‐papaya peels

The pectin sample was obtained from mixed banana–papaya peel powder, which was analyzed using the FTIR spectrometer for identification of functional groups present in it; and it was compared with the commercial pectin (used as standard). The FTIR spectra of the banana–papaya peel pectin and commercial pectin (standard) correspond to the characteristics of functional groups present in the spectrums (Figure 10a,b).

The region centered at about 3419 cm^−1^ and 3436 cm^−1^ of the strong broadband for both commercial and extracted pectin, respectively, agrees to the −OH stretching absorption that is due to inter‐ and intramolecular hydrogen bonds. In pectin samples, an absorption peak is found in the O‐H region, which might be inter‐ and intramolecular hydrogen bonding of the galacturonic acid backbone (Abid et al., 2017). The peaks at 2943 cm^−1^ (in commercial pectin) and 2925 cm^−1^ (in extracted pectin) samples refer to C‐H stretching and bending vibrations of CH, CH_2_, and CH_3_. Regions centered at about 1740 cm^−1^ (strong peak) were observed on both commercial and extracted pectin that describes the carboxylic acid methyl ester functional C = O stretching. The peak noticed around 1445 cm^−1^ in both cases was ascribed for the free carboxyl C = O stretching vibration. These findings confirmed and are well agreed with the result of the “Saba” banana peel pectin using the microwave extraction (MAE) method that was reported (Rivadeneira et al., 2020).

As seen in Figure 10a,b, the “fingerprint” region (ranged: 800–1200 cm^−1^) reveals the indication of chemical groups being present in polysaccharides of the pectin as similar to the pectin that was extracted from the citrus peel, which was reported *(*Jafari et al., 2017; Khamsucharit et al., 2018). Accordingly, the obtained results confirmed that the extracted polysaccharide of this current study is found to be pectin.

### 3.7 Functional attributes of pectin powder

Functional properties are allied to the chemical structure of the polysaccharides. Generally, functional properties of the pectin are dependent on the particle size, process conditions, the structure of polysaccharides, and sources (Yesica et al., 2016). Functional attributes of the studied pectin are shown in Table 6.

**TABLE 6:** Functional properties of mixed banana–papaya peel pectin powder

Properties	Values	Literature	Reference
WAC (g/g)	8.23 ± 2.84	8–10	Lundberg et al. (2015)
OAC (g/g)	3.44 ± 1.27	2.4	Ognyanov et al. (2018)
SC (mL/g)	22.73 ± 0.28	17.1	Nguyen et al. (2015)
EA (%)	45.16 ± 4.08	44.97–47.71	Yang et al. (2018)
ES (%)	29.33 ± 5.13	36.14	Yang et al. (2018)

Note

Data are expressed as mean ± standard deviation (*n* = 3).

Abbreviations: EA, emulsifying activity; ES, emulsion stability; OAC, oil absorption capacity; SC, swelling capacity; WAC, water absorption capacity.

#### 3.7.1 Water and oil absorption capacity

Water absorption capacity (WAC) is the ability of a material to hold the bound, hydrodynamic, capillary, and physically entrapped water (Wongkaew et al., 2020). The WAC of the isolated pectin was found as 8.23 ± 2.84 g water/g sample (Table 6). This is lesser than the WAC (10.8 g/g) obtained from in vitro cultures of *F. officinalis* (Ognyanov et al., 2018), but within (8–10 g/g) the range of WAC of citrus pectin (Lundberg et al., 2015). The difference in WAC of the extracted pectin is due to the available chemical composition on plant material.

The oil absorption capacity (OAC) of the studied banana–papaya peel pectin (3.44 ± 1.27 g/g) was found to be higher than that of (2.4 g oil/g sample) the previously studied pectin which was obtained from in vitro cultures of *F. officinalis*. This is due to raw materials used and extraction conditions employed. It supports the higher potential to absorb oil possessing the greater surface area, makes the thickness of the pectin introduced materials better, and enhances the hydrophobic nature of the pectin; it is proved with the lower WAC of this presently studied pectin.

#### 3.7.2 Swelling capacity

Swelling capacity (SC) is related to the structural features and chemical alignment of the fiber that show a vital part take‐up of water. It offers the amount of fiber lattice inflame when water is ingested (Nguyen et al., 2015). The SC of pectin (dietary fiber) of this study was reported to be 22.73 ± 0.28 ml/g sample. This result was found to be higher than those obtained for other dietary fibers extracted from corn, green beans, and potatoes’ waste reported (Yesica et al., 2016) and other researchers finding in wheat (3.4 ml/g), in apple (7.1 ml/g), and in oat (2.3 ml/g). The greater SC is allied with the quantity of soluble dietary fiber, especially pectin, which can keep more of water and oil part present food product does make the stabilization means to control the phase separation in yogurt products.

#### 3.7.3 Emulsifying properties of pectin

Emulsion properties of pectin are the capacity to produce the emulsion and which are the significant properties for pectin‐like hydrocolloids to use as stabilizers in food products, to facilitate the solubilization or the dispersion of two immiscible liquids, and to sustain the reliability of an emulsion (Pasandide et al., 2018; Yesica et al., 2016). Emulsion activity (EA) and emulsion stability (ES) of the studied pectin were assessed. The EA of the presently studied pectin (at optimized conditions) was found to be 45.16 ± 4.08% (Table 5) and this was found to be in good agreement with the EA of apple pectin (45.34%) and within the range of EA of potato pulp pectin (44.97%–47.71%) reported earlier (Yang et al., 2018).

Similarly, the ES of the presently studied mixed banana–papaya peel pectin emulsions was found as 29.33 ± 5.13%; comparatively, it is lower than the ES of the commercial citrus pectin (36.14%) and higher than that of the apple pectin (19.15%) emulsions. The higher ES for extracted pectin might be due to a higher degree of acetylation and higher sugar side chains of the rhamnogalacturonan (RG‐I) domain proposed earlier (Yang et al., 2018).

### 3.8 Solubility of pectin powder

Solubility inspection (Table 7) has shown that the studied pectin was found to be completely solubilized in hot water, cold alkali, and hot alkali as well as slightly soluble in cold water. But it was found to be insoluble in all of the organic solvents like ethanol, acetone, and methanol used in this study as similar to earlier reports (Khan et al., 2017; Tyagi & Yoges, 2016). This slightly solubilizing (in cold water) effect of the currently studied banana–papaya mixed pectin probably contains the electrolytes that may be hindering the solubility of pectins, may have a slightly higher ash content (6.20 ± 1.12%), and the process (drying) of studied pectin might be the additional cause for slighter solubility of pectin in cold water. This observation was made to be in good agreement with the reported literature (Begum et al., 2014).

**TABLE 7:** Solubility profile of dried pectin powder

Solvents	Solubility
Cold water	Slowly soluble
Hot water	Soluble
Cold alkali (NaOH)	Soluble
Hot alkali (NaOH)	Soluble
Ethanol	Insoluble
Acetone	Insoluble
Methanol	Insoluble

## 4 CONCLUSION

By employing the ANOVA tool and regression model of response surface desirability optimization approach, the optimal parameters for pectin extraction were established in this study upon maximum extraction yield and the AUA amount of pectin from mixed banana–papaya fruit peels was found to be temperature: 73°C, extraction time: 35 min, and pH: 2.0. Based on these optimal conditions, 23.74 ± 0.25% yield and 69.97 ± 0.05% of anhydrouronic acid content were reported in extracted pectin.

Physicochemical properties of the studied pectin at optimized conditions were evaluated as moisture content (7.2%), ash content (6.2%), crude protein (3.92%), methoxyl content (8.37%), degree of esterification (67.91%), equivalent weight (783.69 g/mol), and acetyl value (0.48%); and some functional properties like water absorption capacity (WAC), oil absorption capacity (OAC), swelling capacity (SC), emulsifying activity (EA), and emulsion stability (ES) were assessed and the results were obtained as 8.23%, 18.44%, 22.73%, 45.16%, and 29.33%, respectively). The obtained results of the physicochemical characterization of optimized extracted pectin showing the mixed banana and papaya peel waste are a potential source for the extraction of commercial pectin; and the studied extracted pectin was categorized under high methyl ester pectin with the degree of esterification (67.91 ± 0.33), which was found to be >50% and the accepted measure of pectin purity with the AUA content (69.97 ± 0.05) above 65%. Thus, the studied banana–papaya peel pectin is considered as a food additive for many uses according to the International Pectin Producers’ Association (IPPA).

## ETHICAL APPROVAL

All authors declare that this study does not involve animal and human subjects.

## CONFLICT OF INTEREST

All authors declare that there is no conflict of interest.

## Data Availability

All necessary data are provided in the article. Nevertheless, the authors agree to share raw data upon request.
